# Progressive Lower Extremity Weakness and Axonal Sensorimotor Polyneuropathy from a Mutation in *KIF5A* (c.611G>A;p.Arg204Gln)

**DOI:** 10.1155/2015/496053

**Published:** 2015-10-12

**Authors:** Nivedita U. Jerath, Tiffany Grider, Michael E. Shy

**Affiliations:** Department of Neurology, Carver College of Medicine, University of Iowa, 200 Hawkins Drive, Iowa City, IA 52242, USA

## Abstract

*Introduction*. Hereditary Spastic Paraplegia (HSP) is a rare hereditary disorder that primarily involves progressive spasticity of the legs (hamstrings, quadriceps, and calves). *Methods*. A 27-year-old gentleman was a fast runner and able to play soccer until age 9 when he developed slowly progressive weakness. He was wheelchair-bound by age 25. He was evaluated by laboratory testing, imaging, electrodiagnostics, and molecular genetics. *Results*. Electrodiagnostic testing revealed an axonal sensorimotor polyneuropathy. Genetic testing for HSP in 2003 was negative; repeat testing in 2013 revealed a mutation in KIF5A (c.611G>A;p.Arg204Gln). *Conclusions*. A recent advance in neurogenetics has allowed for more genes and mutations to be identified; over 76 different genetic loci for HSP and 59 gene products are currently known. Even though our patient had a sensorimotor polyneuropathy on electrodiagnostic testing and a 2003 HSP genetic panel that was negative, a repeat HSP genetic panel was performed in 2013 due to the advancement in neurogenetics. This revealed a mutation in *KIF5A*.

## 1. Introduction

Hereditary Spastic Paraplegia (HSP) is a rare hereditary disorder that involves progressive spasticity of the legs (hamstrings, quadriceps, and calves). Pathophysiology of HSP involves a length dependent degeneration of the corticospinal tract, degeneration of longest ascending sensory fibers, degeneration of spinocerebellar fibers, and neuronal cell bodies.

Previous classifications of HSP included age, mode of inheritance, or symptoms. Classifying by age, type 1 HSP is earlier (age < 35 years old) with slower progression; weakness, sensory loss, and urinary symptoms are less marked. Type 2 HSP (age > 35) is later with a more rapidly progressive disease, muscle weakness, sensory loss, and marked urinary involvement. These individuals lose the ability to walk by the age of 60–70. HSP can be inherited AD, AR, or X-linked recessive. HSP can be pure (spasticity in lower limbs alone) or complicated with additional symptoms (peripheral neuropathy, epilepsy, ataxia, optic neuropathy, retinopathy, dementia, ichthyosis, mental retardation, deafness, and problems with speech/swallowing).

Recent classifications of HSP have focused on abnormal cellular function. A recent advance in neurogenetics over the past 10 years has allowed for more genes and mutations to be identified; over 76 different genetic loci and 59 gene products are currently known [1].

We report a case of HSP in a patient who required repeat genetic testing for this rare condition. This case reflects the marked advancement in diagnosis of genetic disorders and the need for repeat genetic testing if prior genetic testing has been outdated. Even though our patient had a sensorimotor polyneuropathy on electrodiagnostic testing and a 2003 HSP genetic panel that was negative, a repeat HSP genetic panel performed in 2013 revealed a mutation in KIF5A resulting in his clinical presentation.

## 2. Case Report

A 27-year-old man presented with progressive weakness in his lower extremities and a slowly developing inability to walk. He was the product of a normal pregnancy and delivery. He reached his developmental milestones on time; he walked at around 1 year of age. He was able to play soccer as a child and was a fast runner until age 9, when he stopped playing soccer. He developed lower extremity weakness after a 2-3-day flu-like illness. His symptoms slowly progressed ever since. For example, he was able to run (although slowly) at age 15 but unable to do so at age 21. He continued to walk until age 25 when he had to use a wheelchair. At age 18, he developed attention deficit hyperactivity syndrome (ADHD) and was started on Dexedrine. He was morbidly obese and developed obstructive sleep apnea for which he was started on a BiPAP (bilevel positive airway pressure).

Review of systems was positive for occasional muscle cramps and muscle stiffness. He had no loss of sensation in the lower extremities, no problems with fine motor function of his hands, no bowel or bladder incontinence, no diabetes, and no scoliosis.

Examination at age of 27 years was significant for distal lower extremity weakness: MRC grade 4/5 strength in the tibialis anterior muscle, gastrocnemii, foot eversion, right foot inversion, and right toe dorsi/plantar flexion. Sensory examination was significant for mild decrease in vibration in toes, but the rest of the sensory exam was normal. Deep tendon reflexes were brisk in his lower extremities with sustained clonus at both ankles. He had bilateral Babinski signs and a brisk brachioradialis reflex on the left. Hoffman's sign was absent. Jaw jerk reflex was normal. He had increased tone and spasticity in both legs.

### 2.1. Laboratory Testing

Vitamin B-12, folic acid, ceruloplasmin, homocysteine, vitamin E, arylsulfatase A, methylmalonic acid, peroxisomal panel, VDRL, HTLV-1, and thyroid testing were normal.

### 2.2. Imaging

Brain and spine MRI were normal.

### 2.3. Electrodiagnostic Testing

Electrodiagnostic testing was performed and results were significant for an axonal sensorimotor polyneuropathy.

### 2.4. Pedigree

**Figure 1:**
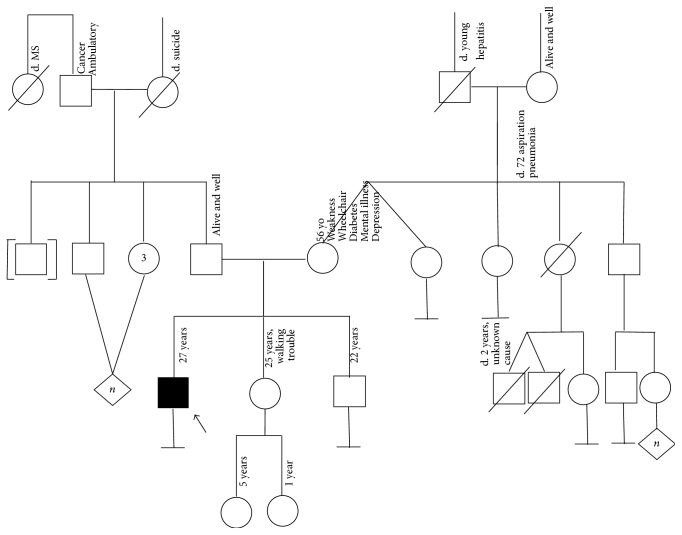
Family pedigree. Squares indicate men; circles indicate women; diagonal lines indicate deceased; black indicates* KIF5A* mutation. An arrow indicates the proband. “*n*” stands for number of unaffected relatives.

A family pedigree was unrevealing except for mom with possible “Charcot Marie Tooth Disease” and being in a wheelchair; the patient's sister had “walking trouble,” which was attributed to previous infection with polio (see Figure 1). Mom and sister chose not to undergo genetic testing.

### 2.5. Genetic Testing in 2003

Genetic testing from a tertiary referral center in 2003 came back with a normal frataxin gene (for Friedreich's ataxia) and normal SPG3A and SPG4 (for Autosomal Dominant Hereditary Spastic Paraplegia 3A and Spastic Paraplegia 4), and the patient was left with no diagnosis.

### 2.6. Genetic Testing in 2013

Given the strong clinical suspicion of Hereditary Spastic Paraplegia (HSP) with predominantly lower extremity spasticity, further genetic testing for HSP was performed via a commercial lab. He had an updated panel of genes for HSP that were tested and came back normal:* BSCL2* (Spastic Paraplegia 17),* KIAA0196* (Spastic Paraplegia 8),* NIPA1* (Spastic Paraplegia 6), and* REEP1* (Spastic Paraplegia 31), plus a negative deletion analysis of* SPAST* and* REEP1*. Genetic testing results for the* KIF5A* gene showed a heterozygous missense mutation—“a variant of unknown significance” (c.611G>A;p.Arg204Gln) per report. Upon more detailed review of the literature, however, this variant was responsible for SPG 10 (Spastic Paraplegia type 10) and axonal CMT type 2 disease [2, 3].

## 3. Discussion

The prevalence of HSP is rare (2–6/100,000). Presentation can occur at any age from infancy to late adulthood (there have been cases reported at age of 85 years). Most patients, however, will experience onset between second and fourth decades of life. Patients with HSP (who have corticospinal tract, dorsal column, and spinocerebellar degeneration) may present with a hereditary motor and/or sensory neuropathy; our patient was found to have an axonal sensorimotor polyneuropathy on electrodiagnostic testing as well as diminished vibration sense on exam.

Our patient presented with lower extremity weakness and spasticity that progressed steadily since childhood. There were upper motor neuron signs (increased muscle tone, brisk reflexes, and upgoing toes) as well as mild sensory loss in lower extremities. Differentials for our patient's presentation include HSP (the official abbreviation is SPG), MTHR deficiency, multiple sclerosis, spinocerebellar ataxia, cervical/lumbar spondylosis, arginase deficiency, vitamin B-12/vitamin E/copper deficiency, lathyrism, HTLV-1, Friedrich's ataxia, Krabbe's disease, ALS, PLS, metachromatic leukodystrophy, or adrenoleukodystrophy (see Table 1). Important negative findings that led to our patient's diagnosis include normal cranial nerve function, no corticobulbar involvement, no autonomic disturbances, no bladder dysfunction, no ataxia, and normal upper extremity function.

Clinically, individuals with HSP will have difficulty walking, increased muscle tone, weakness in the legs (more commonly in iliopsoas, tibialis anterior muscle, and hamstrings), reduced sensation in legs, urinary incontinence, and fatigue due to increased effort or poor sleep from cramps.

Examination features include increased muscle tone, weakness, mild loss of vibratory sensation, and upper motor neuron signs in the lower extremities distinguishing HSP from other diseases. High arched feet are prominent in older patients. MRI of the spinal cord can show atrophy of the spinal cord. Cortical evoked potentials show a reduced conduction velocity and reduced amplitude of the evoked potential of the corticospinal tract. Lower extremity somatosensory evoked potentials (SSEPs) show a conduction delay in the dorsal column fibers. Upper extremity SSEPs are usually normal. CSF protein can be mildly elevated.

Our patient had a mutation in* KIF5A* resulting in SPG 10; this leads to a mutation in kinesin, a two-headed motor protein in eukaryotic cells resulting in abnormal movement along microtubule filaments [4]. Kinesin uses energy derived from ATP hydrolysis to transport diverse types of intracellular cargo towards the ends of microtubules within axons [5]. Kinesin is powered by ATP and supports mitosis, meiosis, and axonal transport. It is involved in anterograde axonal transport (cell body to the periphery), whereas dynein is responsible for retrograde transport. Mutated kinesin decreases the efficiency of cargo transport to the distal axon because the mutated kinesin is slower and has a reduced microtubule binding affinity. The mutation is hypothesized to act in a dominant-negative manner by competing with wild-type motors for cargo binding [6].

Clinical features of SPG 10 have been described in a 2009 paper from France illustrating 17 patients from eight families. 7 out of the 8 families had a complex phenotype with peripheral neuropathy, severe upper limb amyotrophy, mental impairment, Parkinsonism, deafness, and/or retinitis pigmentosa [3]. A study performed in 2014 confirmed that* KIF5A* mutations can involve both the peripheral and central nervous system, resulting in variable phenotypes ranging from HSP to Charcot Marie Tooth Disease type 2 [7]. As discussed in the paper, there were three patients who had CMT2 as the predominant phenotype, but only one had classical CMT2, whereas the other two had some pyramidal tract involvement. The other three discussed in this paper had Spastic Paraplegia with two of them having a peripheral neuropathy; our patient is similar to the ones with Spastic Paraplegia as the predominant phenotype. Additional features of the six previously reported patients included cognitive dysfunction, learning difficulties, and cerebellar ataxia; our patient had ADHD [7].

Treatment of HSP involves physical therapy (exercise and muscle flexibility), orthotics, orthopedic surgical interventions, Botox, dorsal rhizotomy, and baclofen for treatment of spasticity. Further knowledge of kinesin-1 movement and its influence on axon generation may result in therapies for diseases such as SPG 10.

Our case illustrates that clinical findings are crucial and given our patient's significant lower extremity spasticity, the diagnosis of Hereditary Spastic Paraplegia (HSP) was still in favor despite initial negative genetic testing for HSP. Furthermore, it has been discovered that* KIF5A* mutations can involve both the peripheral and central nervous system, resulting in variable phenotypes ranging from HSP to Charcot Marie Tooth Disease type 2.

Given the recent advancement in neurogenetics over the past 10 years, it is important to repeat genetic testing that has been outdated. The initial genetic testing was done in 2003 when the “complete” HSP panel included* SPG3A* and* SPG4*; genetic testing for HSP was repeated in 2013 at a time when many new genes for HSP have been discovered. Additional genetic testing for HSP confirmed the diagnosis of a* KIF5A* mutation in our patient; with strong clinical skills and suspicion and knowledge of appropriate genetic testing, elusive diagnoses are becoming more readily apparent.

## Figures and Tables

**Table 1:** Differential diagnosis of lower extremity weakness and spasticity.

Disease	Upper motor neuron signs	Peripheral neuropathy	Cognitive dysfunction	Cerebellar ataxia	Other
HSP (Hereditary Spastic Paraparesis)	•	•			Bilateral symmetric lower extremity spasticity, gait disturbance, urinary urgency, sparing of craniobulbar function, and abnormal SSEP's

MTHR (methylene tetrahydrofolate reductase) deficiency	•	•	•		Behavior changes, seizures, and leukoencephalopathy

Multiple sclerosis	•			•	Wide range: dysarthria, dysphagia, optic neuritis, nystagmus, chronic pain, fatigue, weakness, and bladder/bowel difficulties

Spinocerebellar ataxia				•	Dysarthria, nystagmus, intentional tremor, and hyporeflexia

Cervical or lumbar spondylosis	•				Neck or back pain, leg or arm weakness, abnormal gait, loss of bowel/bladder control, and MRI imaging of the spine will be abnormal

Arginase deficiency	•		•	•	Seizures and tremor, usually evident by age 3

Vitamin B-12 deficiency	•	•	•		Macrocytic anemia

Vitamin E deficiency		•		•	Retinitis pigmentosa

Copper deficiency	•	•			Optic neuropathy, anemia, and neutropenia

Lathyrism	•				From excessive consumption of the chickling pea; restricted to India, Bangladesh, and Ethiopia; it results in an irreversible, nonprogressive spastic paraparesis

HTLV-1 (human T lymphocytic virus-1)	•	•			More frequent in IV drug users, weakness, nocturia, arthralgia, gingival bleeding, dry oral mucosa, and erectile dysfunction

Friedreich's ataxia		•		•	Usually no spasticity (although it can develop later); pes cavus, scoliosis, cardiomyopathy, and arrhythmias

Krabbe's disease	•	•	•	•	Loss of vision is also seen.

ALS (amyotrophic lateral sclerosis)	•				ALS is typically more rapidly progressive and not limited to legs as seen in HSP. Symptoms include upper and lower motor neuron signs, weakness, fasciculations, cramps, dysarthria, dysphagia, dyspnea, muscle spasms, sialorrhea, emotional lability, and cognitive difficulties.

PLS (primary lateral sclerosis)	•				Not limited to legs as seen in HSP; weakness, dysarthria, dysphagia, emotional lability, and bladder urgency can be seen.

Metachromatic leukodystrophy	•		•		Seizures, optic atrophy, and tremors can be seen.

Adrenal leukodystrophy	•		•	•	Vision loss, seizures, adrenal insufficiency, dysphagia, dysarthria, deafness, weakness, vomiting, or aggression can be seen.

## References

[1] Klebe S., Stevanin G., Depienne C. (2015). Clinical and genetic heterogeneity in hereditary spastic paraplegias: from SPG1 to SPG72 and still counting. *Revue Neurologique*.

[2] Crimella C., Baschirotto C., Arnoldi A. (2012). Mutations in the motor and stalk domains of KIF5A in spastic paraplegia type 10 and in axonal Charcot-Marie-Tooth type 2. *Clinical Genetics*.

[3] Goizet C., Boukhris A., Mundwiller E. (2009). Complicated forms of autosomal dominant hereditary spastic paraplegia are frequent in SPG10. *Human Mutation*.

[4] Blackstone C. (2012). Cellular pathways of hereditary spastic paraplegia. *Annual Review of Neuroscience*.

[5] Fichera M., Lo Giudice M., Falco M. (2004). Evidence of kinesin heavy chain (KIF5A) involvement in pure hereditary spastic paraplegia. *Neurology*.

[6] Ebbing B., Mann K., Starosta A. (2008). Effect of spastic paraplegia mutations in KIF5A kinesin on transport activity. *Human Molecular Genetics*.

[7] Liu Y.-T., Laurá M., Hersheson J. (2014). Extended phenotypic spectrum of *KIF5A* mutations: from spastic paraplegia to axonal neuropathy. *Neurology*.

